# Identification of Quantitative Trait Loci Controlling Ethylene Production in Germinating Seeds in Maize (*Zea mays* L.)

**DOI:** 10.1038/s41598-020-58607-1

**Published:** 2020-02-03

**Authors:** Dongdong Kong, Xiuyi Fu, Xiaohui Jia, Wenhui Wang, Yi Li, Jiansheng Li, Xiaohong Yang, Chuanli Ju

**Affiliations:** 1 College of Life Sciences, Capital Normal University, Beijing, China; 2 Beijing Key Laboratory of Crop Genetic Improvement, National Maize Improvement Center of China, China Agricultural University, Beijing, China; 3 Institute of Pomology, Chinese Academy of Agricultural Sciences, Xingcheng, China

**Keywords:** Agricultural genetics, Development, Quantitative trait, Plant development, Plant hormones

## Abstract

Plant seed germination is a crucial developmental event that has significant effects on seedling establishment and yield production. This process is controlled by multiple intrinsic signals, particularly phytohormones. The gaseous hormone ethylene stimulates seed germination; however, the genetic basis of ethylene production in maize during seed germination remains poorly understood. In this study, we quantified the diversity of germination among 14 inbred lines representing the parental materials corresponding to multiple recombinant inbred line (RIL) mapping populations. Quantitative trait loci (QTLs) controlling ethylene production were then identified in germinating seeds from an RIL population constructed from two parental lines showing differences in both germination speed and ethylene production during germination. To explore the possible genetic correlations of ethylene production with other traits, seed germination and seed weight were evaluated using the same batch of samples. On the basis of high-density single nucleotide polymorphism-based genetic linkage maps, we detected three QTLs for ethylene production in germinating seeds, three QTLs for seed germination, and four QTLs for seed weight, with each QTL explaining 5.8%–13.2% of the phenotypic variation of the trait. No QTLs were observed to be co-localized, suggesting that the genetic bases underlying the three traits are largely different. Our findings reveal three chromosomal regions responsible for ethylene production during seed germination, and provide a valuable reference for the future investigation of the genetic mechanism underlying the role of the stress hormone ethylene in maize germination control under unfavourable external conditions.

## Introduction

Maize (*Zea mays* L.) is one of the most important food crops worldwide and is widely used as genetic research material for studying various traits. Seed germination is a developmental event that is crucial for plant propagation. Uniform seed germination and seedling emergence are prerequisites for high yield production in corn. Germination is initiated following the imbibition of water by quiescent dry seeds and is completed following the protrusion of the radicle through the seed coat and endosperm^1^. This process is regulated by sophisticated endogenous plant components as well as environmental factors^2–6^.

Intensive exploration of the mechanism underlying germination in the model plant Arabidopsis (*Arabidopsis thaliana*) has revealed the pivotal roles of plant hormones and other signalling molecules in this complicated process^2,4,5,7–11^. Although germination in crop species has not received as much attention as that in Arabidopsis, early findings in maize using mutants that are deficient in or insensitive to abscisic acid (ABA)^12–14^ have facilitated the establishment of a major concept in germination: that the balance between ABA and bioactive gibberellins (GA) and not the actual amount of each hormone is the primary factor that determines seed germination vigor^2,5,15,16^. Post-genomic technologies have facilitated recent advances in maize germination studies, and numerous genes associated with seed germination under normal or stress conditions have been identified through transcriptomic, proteomic or metabolic analysis^17–20^. Quantitative trait locus (QTL) mapping, which identifies natural allelic variation for complex quantitative traits, is another strategy used to study seed germination. The investigations of QTLs for four germination vigour-related traits at four time points after artificial ageing treatment in two maize recombinant inbred line (RIL) populations revealed 65 QTLs for seed vigour traits^21^. Studies on the genetic mechanism of seed germination involving various aspects of the trait and using mapping populations with different backgrounds are not only of academic value but also of agronomic significance.

The phytohormone ethylene modulates wide-ranging aspects of plant growth and development, including agronomically important processes such as seed germination and fruit ripening^22–25^. Ethylene promotes seed germination in both Arabidopsis and other species, such as *Lepidium sativum*, sunflower, and *Stylosanthes humilis*, by repressing the inhibitory effects of ABA and weakening the structure of tissues surrounding the seed radicle^4,5,26–29^. Ethylene is the primary regulator involved in the sensing of heterogeneous soil conditions by germinating Arabidopsis seedlings^30^, and it increases Arabidopsis seed germination under salt stress^31^. Ethylene production increases during the germination of cocklebur and *Amaranthus caudatus* seeds^32,33^. Under conditions of high salinity, ethylene production by *Stylosanthes humilis* seeds decreases, and seed germination is inhibited^29,34^. In maize, decreased ethylene production is considered to be one of the mechanisms by which methyl jasmonate inhibits germination^35^, indicating an increase in ethylene production and a positive effect of ethylene on maize germination under normal conditions. However, little is known about the role of ethylene in gemination and the genetic loci that control ethylene production in maize during germination.

The main objective of the current study was to map genome-wide chromosomal regions affecting ethylene production in germinating maize seeds. We first investigated the germination phenotypes of 14 inbred lines corresponding to multiparent populations^36^. Then, we selected an RIL population constructed using two parental lines with different germination speeds and identified QTLs for the production of the important germination regulator ethylene during seed germination. We also examined the potential genetic link between ethylene production and two other traits, seed germination and seed weight, in relation to the germination behaviour of maize seeds.

## Results

### Phenotypic diversity of seed germination in 14 inbred lines

**Figure 1:**
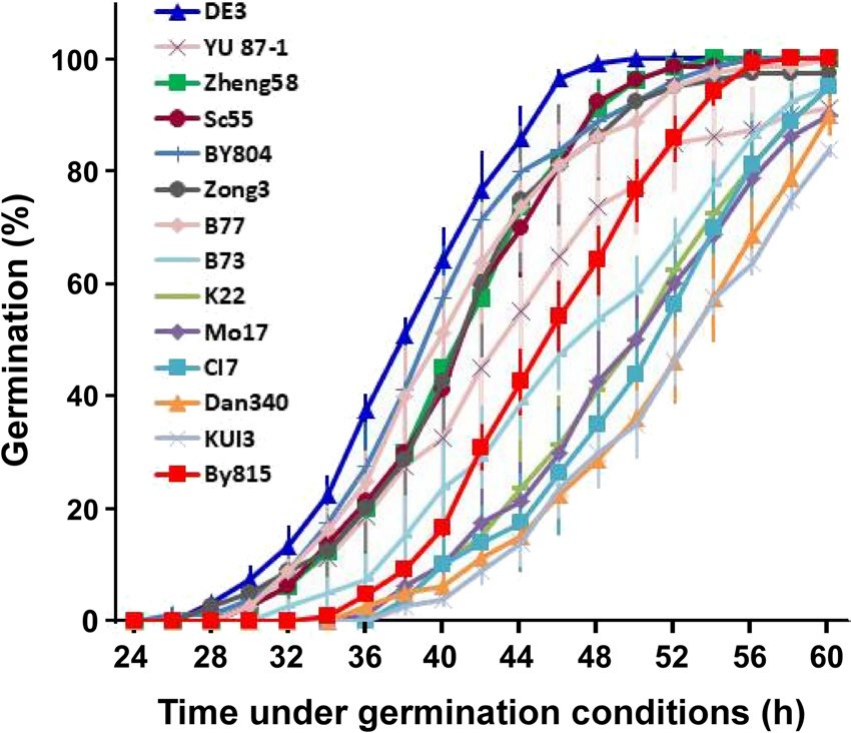
Time-course quantification of seed germination in 14 maize inbred lines. The inbred lines DE3, YU87-1, ZHENG58, SC55, B77, ZONG3, BY804, B73, K22, Mo17, CI7, DAN340, KUI3, and BY815 were incubated under germination conditions for 24–60 h prior to the analysis. The number of germinated seeds was recorded as a percentage (%) of the total number of seeds tested. The results for DE3 and BY815 are shown with blue and red lines, respectively. Significant differences were observed between lines DE3, YU87-1, ZHENG58, SC55, ZONG3, and B77 and lines K22, Mo17, CI7, BY815, DAN340, and KUI3 at the examined time points during the 32–52 h period (*P* < 0.05). For each line, seeds collected from two different years were analysed. For seeds from the same year, two replicates were tested, with 25 seeds in each replicate. The data represent the means ± SEs (n = 4).

To identify suitable maize materials for our study of germination-related mechanisms, we investigated the differences in germination among 14 of 16 inbred lines reported previously^36^. Seeds from two years were analysed, and the correlation of germination in these lines between years ranged from 0.878–0.968, suggesting that germination was dependent mainly on genetic factors. Our time-course analysis revealed distinct germination differences between the 14 inbred lines (Fig. 1), with germination speeds ranging from fast to slow and BY815 and B73 presenting speeds near the centre of the range. Among the inbred lines showing a fast germination speed, DE3 was of special interest because its germination was the most rapid, with approximately one-half of its maximum germination being observed at 36–40 h (Fig. 1). Among the 10 RIL populations^36^, only one population was established from the DE3 inbred line, which was designated the DE3 × BY815 RIL population. Therefore, we focused on the germination of the other parental line of this population, BY815. As shown in Fig. 1, BY815 presented obviously slower germination than DE3, with approximately one-half of its maximum germination occurring at 44–48 h.

### Differences in germination between DE3 and BY815 seeds and ethylene production during germination

**Figure 2:**
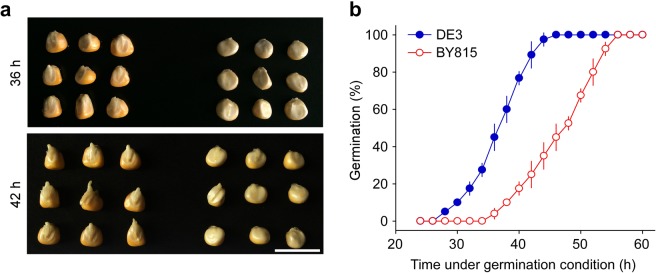
Germination of DE3 and BY815 seeds. (**a**) Phenotypes of DE3 and BY815 seeds after incubation under germination conditions for 36 h (top panel) or 42 h (bottom panel). Three technical repeats were conducted, and representative images are shown. Scale bar = 2 cm. (**b**) Time-course analysis of germination in DE3 and BY815 seeds. Seeds were incubated under germination conditions for 24–60 h, and the number of germinated seeds was recorded as a percentage (%) of the total number of seeds tested. Significant differences were observed between the two samples at all time points during the 30–52 h period (*P* < 0.01). For each sample, 25 seeds were tested in one experiment. The data represent the means ± SDs (n = 3).

We further surveyed the differences in the germination phenotype between DE3 and BY815. Obvious morphological differences were observed between the two lines during germination, with DE3 exhibiting faster radicle appearance and growth than BY815 after 36, 42, and 56 h of incubation under germination conditions (Figs. 2a and S2). Time-course analysis showed that the proportion of germinated DE3 seeds increased from 10% to 90% during the 30–42 h time period after incubation under germination conditions, while the proportion of germinated BY815 seeds increased from 10% to 90% during the 38–54 h time period (Fig. 2b). These data demonstrate the difference in the seed germination speed between maize inbred lines DE3 and BY815, with the former germinating more rapidly than the latter.

**Figure 3:**
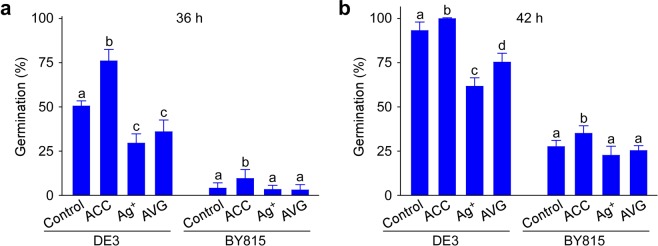
Effects of ethylene reagents on the germination of DE3 and BY815 seeds. Seeds were cultivated in distilled water (the control), 100 µM ACC, 100 µM silver nitrate (Ag^+^), or 10 µM AVG for 36 h (**a**) or 42 h (**b**) under germination conditions, and the number of germinated seeds was recorded as a percentage (%) of the total number of seeds tested. Twenty-five seeds per genotype in each treatment were examined. The data represent the means ± SDs (n = 3), and different letters above the bars indicate significant differences between the treatments (*P* < 0.05).

To gain insight into the ability of ethylene to modulate maize germination, we investigated the effects of the ethylene precursor ACC, the ethylene biosynthesis inhibitor aminoethoxyvinylglycine (AVG)^37^, and silver ions (Ag^+^), which repress ethylene perception^38^, on the germination of DE3 and BY815 seeds at previously reported concentrations^39^. In comparison with the control, ACC stimulated the germination of DE3 and BY815 seeds at both the 36 h and 42 h time points, whereas AVG or Ag^+^ treatment repressed seed germination in the seeds of both lines (Fig. 3). These results show that ethylene positively regulates maize seed germination.

Next, we measured ethylene production in germinating DE3 and BY815 seeds. Determinating a time point that is suitable for the measurement of ethylene production is important. After 42 h of incubation under germination conditions, approximately 85% of the seeds of DE3, the fast-germinating inbred line, had completed germination, while only 25% of the seeds of BY815, the slow-germinating inbred line, had completed germination (Figs. 2b and 3b); therefore, we chose the 36 h time point, when approximately 55% of DE3 seeds had not yet germinated and BY815 seeds (<10% of the seeds had germinated) were about to enter a period of rapidly increasing germination (Fig. 2b), for the measurement of ethylene production. Our results showed that DE3 seeds released more ethylene gas than the BY815 seeds (approximately 2-fold) (Table 1), which was consistent with the faster germination phenotype of DE3 compared with BY815 (Fig. 2). It was notable that under the experimental conditions designed for ethylene production measurement, the germination speeds of the two samples were comparable with the rates observed under normal germination conditions (Table 1; Fig. 2). This suggests the appropriateness of our experimental settings for ethylene production and germination analyses. Taken together, these results show that DE3 and BY815 seeds presented differences in the seed germination speed and produced different amounts of ethylene gas during germination.

**Table 1:** Traits related to ethylene production in germinating seeds, seed germination, and seed dry weight in the two parental lines and the DE3 × BY815 RIL population.

Traits	DE3	BY815	RILs	
	Mean ± SD	Mean ± SD	Mean	Range
Ethylene production (nL/g/12 h)	13.14 ± 2.31	6.56 ± 1.20	9.39	0.29–80.22
Seed germination (%)	43.33 ± 2.89	6.67 ± 2.89	22.21	5.0–72.5
Seed weight (g)	2.68 ± 0.05	2.74 ± 0.08	3.28	1.17–6.95

The dry weight of the seeds was measured before seed imbibition, ethylene production in germinating seeds was measured at the 36 h time point under germination conditions after 12 h of the collection of ethylene gas in each vial, and seed germination was examined immediately after the measurement of ethylene production.

### Phenotypic variation in ethylene production, seed germination, and seed weight in the DE3 × BY815 RIL population

We next analysed seed germination and ethylene production in the DE3 × BY815 RIL population. Since seed germination may be correlated with or affected by seed mass^40^, we also measured the weight of the examined batches of RIL seeds. The correlation coefficients between the two replications of our data were 0.877 for seed germination, 0.962 for ethylene production, and 0.960 for seed weight. A wide range of phenotypic variation was observed for each of the three traits examined (i.e., ethylene production, seed germination, and seed weight) (Table 1), and a roughly normal distribution was shown (Fig. S3). The mean ethylene production in germinating seeds in the RIL population was 9.39 nL/g/12 h, which was close to the mid-parent value of 9.85 nL/g/12 h (Table 1). The mean proportion of seed germination in the RIL population at the 36 h time point was 22.21%, which was slightly lower than the mid-parent value of 25%. The mean seed weight per 20 seeds in the RIL population was 3.28 g, which was higher than the values in both parental lines (DE3 2.68 g and BY815 2.74 g) (Table 1).

To understand the phenotypic relationships between the three traits tested, we conducted Pearson correlation analysis. A moderate negative correlation was observed between seed germination and seed weight (r = −0.359 at *P* ≤ 0.01), whereas no significant correlations were found between ethylene production and seed germination or seed weight (Table 2).

**Table 2:** Pearson correlation coefficients for three traits detected in the DE3 × BY815 RIL population.

	Ethylene production	Seed germination
Seed germination	0.097	
Seed weight	0.000	−0.359**

**The correlation between the traits is significant at the 0.01 level.

### Identification of QTLs for ethylene production in germinating seeds, seed germination, and seed weight

Using QTL Cartographer software, we detected three putative QTLs for ethylene production in germinating seeds, three putative QTLs for seed germination, and four putative QTLs for seed weight in the DE3 × BY815 RIL population (Table 3). According to the 1,000-permutation test results (significant at α = 0.05), the LOD threshold values for the QTLs were defined as 3.1 for ethylene production, 3.4 for seed germination, and 3.4 for seed weight. The genetic intervals of these QTLs ranged from 2.1 to 10.2 centimorgans (cM), with an average value of 5.6 cM.

**Table 3:** List of putative QTLs for three traits detected in the DE3 × BY815 RIL population.

QTL^a^	Chr	Flanking markers	Genetic interval (cM)	Physical position (Mb)^b^	LOD	A^c^	*R*^2^(%)^d^
**Ethylene production**							
*qETH3*	3	PZE-103184341–PZE-103185983	173.21–180.91	229.1–230.3	3.5	4.02	8.3
*qETH6*	6	PUT-163a-94473612–4863–PZE-106006254	6.5–9.71	6.3–8.0	5.0	−5.13	13.2
*qETH9*	9	PZE-109090062–SYN32366	89.81–95.91	137.8–140.8	3.8	4.66	9.2
**Seed germination**							
*qGER3*	3	PZE-103127574–PZA00494.2	99.5–103.3	184.4–187.9	3.8	−4.22	7.0
*qGER6*	6	PZE106014750–PZE-106010000	15.3–19.3	13.0–28.1	3.5	5.34	6.6
*qGER8*	8	PZE-108008327–SYN18173	30.6–35.0	8.4–8.8	3.6	−4.08	6.6
**Seed weight**							
*qWEI1*	1	PUT-163a-71311320–3113–PZE-101035008	50.8–61.0	16.8–22.6	5.0	0.32	8.3
*qWEI2*	2	PZE-102032169–PZE-102034918	47.7–49.8	15.0–16.5	6.2	−0.36	10.3
*qWEI5*	5	PZE-105034878–SYN13829	72.2–78.7	19.8–33.8	3.6	−0.27	5.8
*qWEI9*	9	PZE-109012535–SYN20433	47.5–58.0	13.0–20.3	4.5	0.30	7.3

^a^The identified QTLs. The name of each QTL includes the details of the trait abbreviation and chromosome number.

^b^The physical positions of putative QTL intervals with reference to the B73 reference genome sequence (www.maizesequence.org).

^c^Additive effect of each QTL detected, with positive values indicating that the allele from BY815 increased the trait value and negative values indicating that the allele from DE3 increased the trait value.

^d^The percentage of phenotypic variation explained by individual QTLs.

Among the three QTLs that controlled ethylene production in germinating seeds, *qETH3* was located on chromosome 3, and the other two QTLs, *qETH6* and *qETH9*, were located on chromosome 6 and chromosome 9, respectively (Table 3). These QTLs contributed between 8.3% and 13.2% of the phenotypic variation in ethylene production, with the QTL on chromosome 6 (*qETH6*, LOD = 5.0, *R*^2^ = 13.2%) being the most significant (Table 3). *qETH6* presented alleles associated with increased ethylene production derived from DE3 (Table 3), the high-value parental line in terms of ethylene production during germination (Table 1). QTLs *qETH3* and *qETH9* both exhibited alleles from BY815 that were correlated with increased ethylene production (Table 3).

The three QTLs controlling seed germination were located on chromosomes 3, 6, and 8 and each accounted for 7.0% or 6.6% of the phenotypic variation in the trait (Table 3). The DE3 alleles at two QTLs, *qGER3* and *qGER8*, were associated with increased seed germination, and the BY815 allele at QTL *qGER6* was associated with increased seed germination (Table 3).

Each of the four QTLs controlling seed weight contributed between 5.8% and 10.3% of the observed phenotypic variation, with the QTL located on chromosome 2 (*qWEI2*, LOD = 6.2, *R*^2^ = 10.3%) being the most significant (Table 3). The DE3 allele at QTL *qWEI2* was associated with a phenotypic increase in seed weight (Table 3). QTL *qWEI1* located on chromosome 1, explained 8.3% of the phenotypic variation in the trait and exhibited alleles that increased seed weight from BY815 (Table 3). QTL *qWEI5*, localized on chromosome 5, explained 5.8% of the phenotypic variation of the trait and exhibited alleles from DE3 that increased seed weight (Table 3). QTL *qWEI9* was located on chromosome 9 and had an additive effect due to the BY815 allele (Table 3).

We next analysed whether any of the QTLs for different traits colocalized. Among the ten QTLs detected, four were located on different chromosomes. We then focused on the remaining six QTLs, which clustered in pairs on individual chromosomes. For the two QTLs on chromosome 3, one QTL, for ethylene production (*qETH3*), spanned the 173.21–180.91 cM genetic interval, and the other, for seed germination (*qGER3*), spanned 99.5–103.3 cM (Table 3). For the QTLs located on chromosome 6, *qETH6* was located at 6.5–9.71 cM, and *qGER6* was located at 15.3–19.3 cM (Table 3). For the QTLs on chromosome 9, *qETH9* was located around the 89–96 cM genetic interval, and *qWEI9* was located at 47.5–58 cM (Table 3). In summary, among all QTLs identified, we observed no colocalized chromosomal regions that controlled the traits of ethylene production during germination, seed germination, and seed weight.

## Discussion

In this study, through a combination of physiological, pharmacological, and molecular approaches, we analysed the genetic basis of ethylene production in germinating maize seeds. To our knowledge, this is the first genetic study to examine the amount of the gaseous hormone ethylene generated during germination in a crop mapping population. Because ethylene modulates plant development as well as stress responses^22–25^, the current study performed under normal conditions may provide a useful reference for future investigations of the role of ethylene in crop germination control under unfavourable environmental conditions.

We initiated our study by evaluating of the germination phenotypes of 14 inbred lines. These lines were selected from an association mapping panel consisting of germplasms collected worldwide that represent a wide range of genetic diversity^41^; thus, they may exhibit phenotypic variation for different traits including germination. Furthermore, these lines are the parental lines of 10 individual segregating populations, all of which have been genotyped using the maize SNP50 array^36^, providing multiple options for the subsequent QTL analysis. We found that these lines exhibited phenotypic variation in germination speed (Fig. 1), and we then selected DE3 and BY815, which showed obvious differences in germination speed (Fig. 2) for further investigation. Both lines responded to ethylene-modifying agents (Fig. 3), which suggested that endogenously produced ethylene and ethylene signals play a positive role in maize seed germination. Furthermore, the two lines emitted different amounts of ethylene during germination (Table 1). We therefore used the segregating RIL population established from these two lines to map QTLs for seed germination and ethylene production during germination.

A recent study showed that, even at low levels (tens of nL/L), ethylene stimulates kiwifruit ripening^42^. This finding demonstrates the critical role of trace amounts of ethylene in plant physiology and, thus, the importance of ethylene production monitoring. Different methods have been developed for ethylene monitoring in plants, among which laser-based ETD-300 detectors have only recently been developed and commercialized^43^. This highly sensitive system shows a detection range of 300 pL/L to 300 µL/L at a time-scale of seconds. Moreover, together with a gas-handling system consisting of mass flow controllers and electronic valves, this detector enables the use of three operation modes for ethylene monitoring: continuous flow, stop-and-flow, and sampling^43^. Due to advantages of showing high sensitivity and a rapid response time, the ETD-300 ethylene detector has been applied for studies of different plant species in relation to various research themes, such as development, signalling, abiotic stress and post-harvest physiology^44–54^. Based on the variation in ethylene production that we detected in the germinating DE3 and BY815 seeds using the stop-and-flow mode (Table 1), the ETD-300 detector was chosen as an ideal system for the analysis of the genetic architecture of ethylene production in the DE3 × BY815 RIL population.

QTLs for seed germination have been reported in *Medicago truncatula*, *Brassica oleracea*, sunflower (*Helianthus annuus*), and rice (*Oryza sativa*)^55–58^. Despite the importance of seed germination in maize, few studies on the underlying genetic mechanisms have been conducted in this species. In this study, we identified three QTLs for seed germination and three QTLs for ethylene production in germinating seeds in the DE3 × BY815 RIL population (Table 3). Individually, these QTLs accounted for 6.6%–13.2% of the phenotypic variation of the trait, which is comparable to the effect size of QTLs for seed vigour-related traits, germination energy (GE) and the germination percentage (GP) reported in two other maize RIL populations, which ranged between 5.61% and 10.67%^21^. On the basis of a seed vigour-related study, Han *et al*. reported the detection of sixty-five QTLs for four traits, GE, GP, root dry weight (RDW), and seedling dry weight (SDW), at four time points (0, 2, 4, and 6 days) after artificial ageing treatment, and those QTLs were further clustered into eighteen meta-QTLs (mQTLs)^21^. Interestingly, *qGER3* (physical interval 184.4–187.9 Mb), one of the three QTLs for seed germination identified in our study, was found to overlap partially with mQTL3–4 (physical interval 175.5–184.7 Mb), which contained a cluster of three QTLs for GE and one QTL for GP reported by Han *et al*.^21^. This finding suggests the importance of this region on chromosome 3 in maize seed germination control.

We investigated the genetic relationship between the traits of seed germination and ethylene production in germinating seeds by analysing colocalization of the detected QTLs. Based on our demonstration that ethylene stimulated maize seed germination (Fig. 3), we used an RIL population constructed with DE3 seeds (showing fast germination and high ethylene production) and BY815 seeds (slowing germination and low ethylene production) (Table 1) for the experiment. However, among the six QTLs detected, no overlapping QTLs (i.e., no genetic association) was found between ethylene production and seed germination, which was consistent with the Pearson correlation analysis results showing no statistical correlation between the two traits (Table 2). The absence of QTL colocalization between the two traits could be attributed in part to the small number of QTLs detected for each trait. Further studies involving the monitoring of germination and ethylene production at multiple time points using the continuous flow operation mode of the ETD-300 ethylene detector for ethylene production measurement may help to clarify the genetic association between the traits. Overall, our findings suggest marked differences in the genetic mechanisms controlling seed germination and ethylene production in germinating seeds under our experimental conditions.

Considering the possible correlation between seed mass and germination^40^, we investigated the genetic mechanism of seed weight and analysed its correlation with germination in our samples. We examined the trait of seed weight using the same batch of samples in which we analysed germination. Four QTLs for seed weight were detected (Table 3), and we then compared their locations with the QTLs for ear traits reported in the DE3 × BY815 RIL population^59^. We found that *qWEI5* (physical interval 19.8–33.8 Mb) was colocalized with a QTL for cob weight (physical interval 15.8–22.9 Mb) reported by Xiao *et al*. on chromosome 5^59^ and that *qWEI1* (physical interval 16.8–22.6 Mb) was located near a QTL for cob weight (physical interval 14.1–16.5 Mb) reported on chromosome 1. The finding that two out of the four QTLs for seed weight that we identified overlapped with or were located close to QTLs for ear traits reported in the same RIL population suggests that the seeds that we used for germination analysis represented the population well. We observed a moderate negative correlation between seed germination and seed weight (Table 2), which indicates that seeds with heavy weight tend to germinate slowly.

One of the ultimate goals of QTL mapping is to clone the genes and understand the molecular mechanisms underlying their effects. Ethylene biosynthesized in plants occurs via a well-defined pathway in which ACC synthase (ACS) catalyses the conversion of S-adenosyl-L-methionine (SAM) to ACC, which is then converted to ethylene by ACC oxidase (ACO)^37^. ACS-catalysed ACC formation is thought to be the primary regulatory step in ethylene biosynthesis^60^. Based on the maize B73 reference genome sequence (Version 5b.60) (http://ensembl.gramene.org/Zea_mays/Info/Index)^61^, we compared the physical positions of *ACSs* and *ACOs* with the QTL intervals discovered in this study. We found that *GRMZM2G164405*, which encodes maize ACS2 and is located at 15.8 Mb on chromosome 2, was located precisely in the centre of QTL *qWEI2* (physical interval 15.0–16.5 Mb) and *GRMZM2G018006*, which encodes maize ACS3 and is located at 23.9 Mb on chromosome 9, was located near QTL *qWEI9* (physical interval 13.0–20.3 Mb). In summary, although no genetic loci for ethylene production detected under our experimental conditions were located together or close to *ACSs* or *ACOs*, we observed that two QTLs for seed weight were located close to ACS genes. Although this may be a coincidence, our observations indicate the possibility that variation among ACS alleles may affect seed weight. Further detection of QTLs for ethylene production at multiple time points and under different germination conditions may facilitate the discovery of candidate ethylene biosynthesis genes involved in maize seed germination.

With the advent of new technologies, the process of germination can now be profiled globally at the RNA, protein, and metabolite levels. The combination of these studies with the genome-wide QTL mapping of germination-related trait(s), for example, by analysing the chromosomal positions of the genes revealed by -omic approaches together with the further dissection of the functions of potential candidate genes, will greatly accelerate the progress of research into the mechanisms underlying germination. Breeding programmes that exploit the molecular markers associated with seed germination as well as the application of treatment procedures such as seed priming, which stimulates the pre-germinative metabolism and thereby increases germination^62^, will ensure uniform seed germination and thereby enhance corn yield.

## Materials and Methods

### Plant materials

Fourteen (DE3, YU87-1, ZHENG58, SC55, B77, ZONG3, BY804, B73, K22, Mo17, CI7, DAN340, KUI3, and BY815) of the 16 inbred maize lines used to construct 12 multiple-parent populations reported previously^36^ were used for the germination assay, and the F7 RIL population derived from a cross between DE3 (female parent) and BY815 (male parent) in the F1 generation^36^ was used for QTL mapping in this study. All the genotypes were sown at Sanya, Hainan Province, China, in the winter of 2015, harvested in the spring of 2016, and then used in experiments in 2016. Seeds of the 14 inbred lines were also sown and harvested in Beijing, China, in 2019 and were tested in the experiments in 2019. For the germination assays of the 14 inbred lines, seeds from 2016 and 2019 were tested, with 25 seeds per line being randomly picked from the healthy seeds tested in one experiment and two technical replicates being performed for the seeds from each year. The germination of DE3 and BY815 seeds was examined three additional times, with 25 seeds per sample in each treatment. For the evaluation of the traits of ethylene production, seed germination, and seed weight in the DE3 × BY815 RIL population (207 individual lines) and the two parental lines, 20 seeds per line per sample that were randomly picked from the healthy seeds were examined in one experiment with two technical replicates.

### Germination assays of the 14 inbred lines

The seeds were surface-sterilized as described previously^63^, wiped dry with paper towels, and then stored in gas-tight containers with silica gel beads for three days before the experiments. For the germination assays of the 14 inbred lines under normal conditions or DE3 and BY815 under chemical treatment, the seeds were immersed in distilled water, 100 µM 1-aminocyclopropane-1-carboxylic acid (ACC) (Sigma–Aldrich, St. Louis, MO), 100 μM silver nitrate (Ag^+^) (Sigma–Aldrich), or 10 μM aminoethoxyvinylglycine (AVG) (Sigma–Aldrich) in Petri dishes containing two sheets of moist filter papers on the bottom and two layers of wet gauze on top of the seeds. The seeds were germinated in a growth chamber (28 °C, constant light) for seed imbibition, and germination was assessed based on the visual identification of radicle protrusion (approximately 1 mm) at the indicated time points. The number of germinated seeds was recorded as a percentage (%) of the total number of seeds tested.

### Methods for the evaluation of three traits in the DE3 × BY815 RIL population and the two parental lines

The seeds were surface-sterilized as described previously^63^, wiped dry with paper towels, and then stored in gas-tight containers with silica gel beads for three days before the experiments. For each sample, the weight (g) of 20 dry seeds was determined. The seeds were then placed in a glass vial (20 mL volume), and sufficient water was added to submerge the seeds for imbibition. After the incubation of the seeds in the growth chamber (28 °C, constant light) for 24 h, the vials were sealed with airtight covers and cultivated in the growth chamber for another 12 h (from 24 to 36 h after seed imbibition). Ethylene production was measured using an ETD-300 laser-based photoacoustic ethylene detector (Sensor Sense B.V, Nijmegen, the Netherlands)^64^, which was connected to the sample vials through syringe needles. An image showing the samples incubated in the airtight vials before ethylene monitoring is shown in Fig. S1. The moisture and CO_2_ present in each gas sample were removed prior to the analysis, and ethylene production was detected in stop-and-flow mode with a constantly purified air flux of 3 L/h. The data were analysed using Valve Controller software and are presented as nL/g/12 h. Immediately after the measurement was performed, each vial was opened, and the number of germinated seeds was recorded as a percentage (%) of the total number of seeds tested.

### Phenotypic data analysis and composite interval QTL mapping

Student’s *t* test was performed to analyse the statistical significance of the data. The frequency distribution of each trait and the correlation coefficients between the three traits in the RIL population were analysed using SPSS statistical software (SPSS, Inc., IL, USA).

Single nucleotide polymorphism (SNP)-based high-resolution genetic linkage maps were constructed for the DE3 × BY815 RIL population, which contained 13,729 molecular markers and covered a distance of 1806.4 cM distance in total, with an average genetic length of 0.13 cM between successive markers^36^. We then used this map for QTL analysis of the traits tested in the current study via the composite interval mapping (CIM) method^65^ with Windows QTL Cartographer (version 2.5, model 6) software^66^. The entire maize genome was scanned for QTLs with the setting of a 0.5 cM scanning interval between markers and a 10 cM window size using the forward stepwise regression method with five background control markers. The threshold value for declaring putative QTLs for each trait (α = 0.05) was determined through permutation testing (1,000 times)^67^, and the confidence interval of each QTL was set at the 1-logarithm of odds (1-LOD) value distance from the peak marker^68^.

## Supplementary information

Supplementary information.
