# Supplementary material for: Identification of Quantitative Trait Loci Controlling Ethylene Production in Germinating Seeds in Maize (Zea mays L.)
Source: Sci Rep. 2020 Feb 3;10:1677. doi: 10.1038/s41598-020-58607-1 (PMC6997408; doi:10.1038/s41598-020-58607-1)
Supplement: Supplementary file 1 — Supplementary information. [file 41598_2020_58607_MOESM1_ESM.pdf]

Identification of Quantitative Trait Loci Controlling Ethylene Production in Germinating Seeds in Maize (*Zea mays* L.)

Dongdong Kong[1], Xiuyi Fu[2], Xiaohui Jia[3], Wenhui Wang[3], Yi Li[1], Jiansheng Li[2], Xiaohong Yang[2*] and Chuanli Ju[1*]

[1] College of Life Sciences, Capital Normal University, Beijing, China
[2] Beijing Key Laboratory of Crop Genetic Improvement, National Maize Improvement Center of China, China Agricultural University, Beijing, China
[3] Institute of Pomology, Chinese Academy of Agricultural Sciences, Xingcheng, China

Correspondence:
Dr. Chuanli Ju
chlju@cnu.edu.cn
Dr. Xiaohong Yang
yxiaohong@cau.edu.cn

Supplementary information

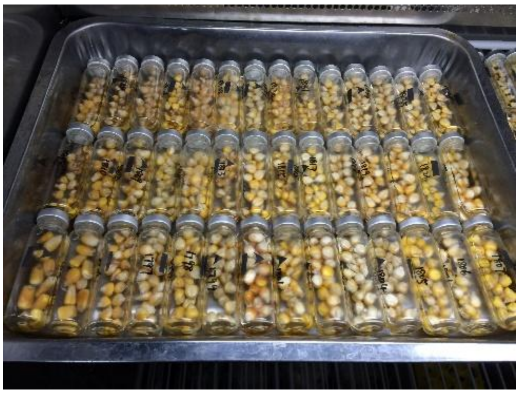

Supplementary Figure S1. The maize samples incubated in air-tight vials in the growth chamber before ethylene monitoring.

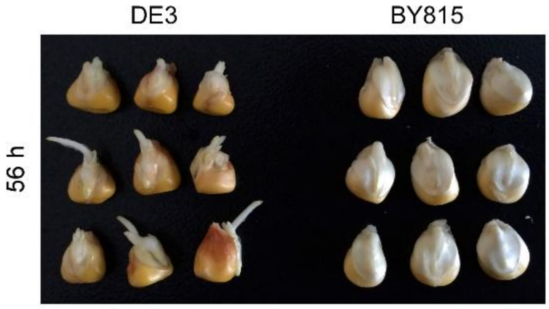

Supplementary Figure S2. Germination phenotypes of DE3 and BY815 seeds after incubation under germination conditions for 56 h.

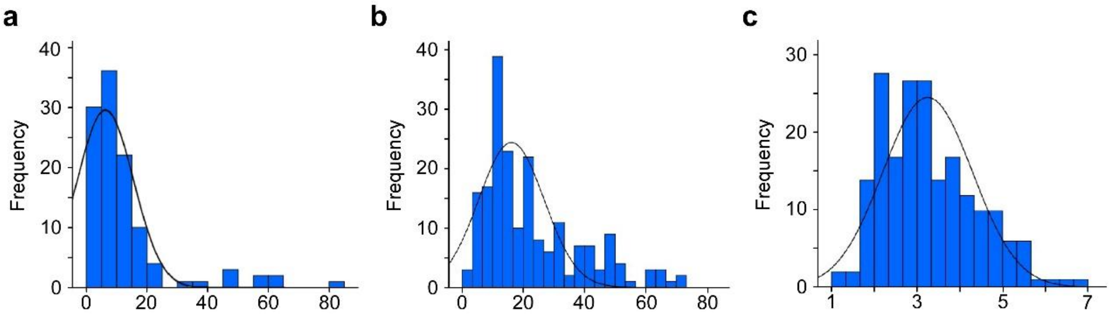

Supplementary Figure S3. Frequency distributions of the traits of ethylene production in germinating seeds (a), seed germination (b), and seed weight (c) in the RIL population.
